# Supplementary material for: Epidemiological modeling of SARS-CoV-2 in white-tailed deer (Odocoileus virginianus) reveals conditions for introduction and widespread transmission
Source: PLoS Comput Biol. 2024 Jul 12;20(7):e1012263. doi: 10.1371/journal.pcbi.1012263 (PMC11268674; doi:10.1371/journal.pcbi.1012263)
Supplement: S1 File — (DOCX) [file pcbi.1012263.s001.docx]

**S1 File. Expert elicitation methods**

From September – December 2022, these two expert panels responded to two sets of questions specific to their discipline and each met for two group discussions. The first set of questions were training questions focused on parameters already estimated in the literature, with the intent of familiarizing experts with the elicitation Shiny platform [1] and the four-point elicitation process and building expert confidence in their ability as an individual and group to capture a likely range of parameter values.

After answering and discussing the training questions, experts answered questions on the parameters of interest. After submitting initial estimates, each expert panel met to facilitate knowledge sharing and discuss clarifications to reduce linguistic uncertainty. Estimates from the four-point elicitation process were fit to a log-normal or logit-normal distribution depending on the nature of each parameter. Following these discussions, experts were then invited to submit revised estimates.

Using finalized parameter estimates for each expert, we fit a log-normal or logit-normal distribution to each response and then averaged across common quantiles in R [2,3]. Lastly, we fit an aggregate distribution using the *qmedist*() function from the fitdistrplus package [4].

Any use of trade, firm, or product names is for descriptive purposes only and does not imply endorsement by the U.S. Government.

References:

1. Chang, W., Cheng, J., Allaire, J., Sievert, C., Schloerke, B., Xie, Y., Allen, J., McPherson, J., Dipert, A. and Borges, B., 2023. shiny: Web application framework for R. R package version 1.7.4.9002, <https://shiny.rstudio.com/>.
2. Howerton, E., Runge, M.C., Bogich, T.L., Borchering, R.K., Inamine, H., Lessler, J., Mullany, L.C., Probert, W.J., Smith, C.P., Truelove, S. and Viboud, C., 2023. Context-dependent representation of within-and between-model uncertainty: Aggregating probabilistic predictions in infectious disease epidemiology. Journal of the Royal Society Interface, 20(198), p.20220659.
3. R Core Team 2023. R: A language and environment for statistical computing. R Foundation for Statistical Computing, Vienna, Austria. https://www.R-project.org/.
4. Delignette-Muller, M. L. and Dutang, C., 2015. fitdistrplus: An R package for fitting distributions. Journal of Statistical Software, 64(4), 1-34. DOI 10.18637/jss.v064.i04.
