# Supplementary material for: Epidemiological modeling of SARS-CoV-2 in white-tailed deer (Odocoileus virginianus) reveals conditions for introduction and widespread transmission
Source: PLoS Comput Biol. 2024 Jul 12;20(7):e1012263. doi: 10.1371/journal.pcbi.1012263 (PMC11268674; doi:10.1371/journal.pcbi.1012263)
Supplement: S2 File — (DOCX) [file pcbi.1012263.s002.docx]

**S2 File: Descriptions of settings for Deer Ecology panel expert elicitation**

Three scenarios were described to experts serving on the Deer Ecology expert panel to contextualize the questions presented to them. This contextualization was essential for reducing linguistic uncertainty within and between experts.

*Setting 1: Wild deer in a rural setting (Questions 4-8, informing Figures S4-S8)*

Consider a wild deer population with the following characteristics to answer the following question. This free-ranging population has a density of 7.7 deer/km^2^ (20 deer/mi^2^). During fall months (September-December) a deer will have an average of 17 proximity events with other wild deer each day. The habitat is typical of the agro-forested midwestern region of the United States, comprised of 40-54% agriculture and 46-60% forested.

Assume that human density in this scenario is approximately 3.1 humans/km^2^, or 8 humans/mi^2^ (typical median density for a rural midwestern U.S. area). Assume that regulated hunting occurs and that hunters use typical harvest methods, including still- and stand/blind based hunting. Baiting and backyard feeding is illegal but may still occur. We define a “proximity event” as an instance in which two deer come within 1.5 m of each other (or a human and a deer come within 1.5 m of each other.) We define “direct contact” as a condition in which two deer make direct physical contact, including mucous membrane contact (through licking, grooming, or mating).

*Setting 2: Wild deer in a suburban setting (Questions 9-10, informing Figures S9-S10)*

Now consider a wild population of deer in a suburban setting. This free-ranging population has a density of 7.7 deer/km^2^ (20 deer/mi^2^). Assume this population shares the landscape with a human population of 100 humans/km^2^, or 259 humans/mi^2^.

We define a “proximity event” as an instance in which two deer come within 1.5 m of each other (or a human and a deer come within 1.5 m of each other.) We define “direct contact” as a condition in which two deer make direct physical contact, including mucous membrane contact (through licking, grooming, or mating).

*Setting 3: Captive deer in a intensive facility setting (Questions 11-13, informing Figures S11-S13)*

Consider a captive deer facility used for captive breeding, meat production, or exhibition, with supplemental feeding and periodic corralling and herding of deer to move between areas for facility operations. These herds are stocked at higher densities relative to wild herds or captive herds kept for hunting opportunities. Human interactions with deer range from the movement of deer between paddocks or facilities, direct handling of deer for breeding purposes and veterinary care, and direct handling by visitors.

We define a “proximity event” as an instance in which two deer come within 1.5 m of each other (or a human and a deer come within 1.5 m of each other.)
