# Supplementary figures and images for: Epidemiological modeling of SARS-CoV-2 in white-tailed deer (Odocoileus virginianus) reveals conditions for introduction and widespread transmission
Source: PLoS Comput Biol. 2024 Jul 12;20(7):e1012263. doi: 10.1371/journal.pcbi.1012263 (PMC11268674; doi:10.1371/journal.pcbi.1012263)

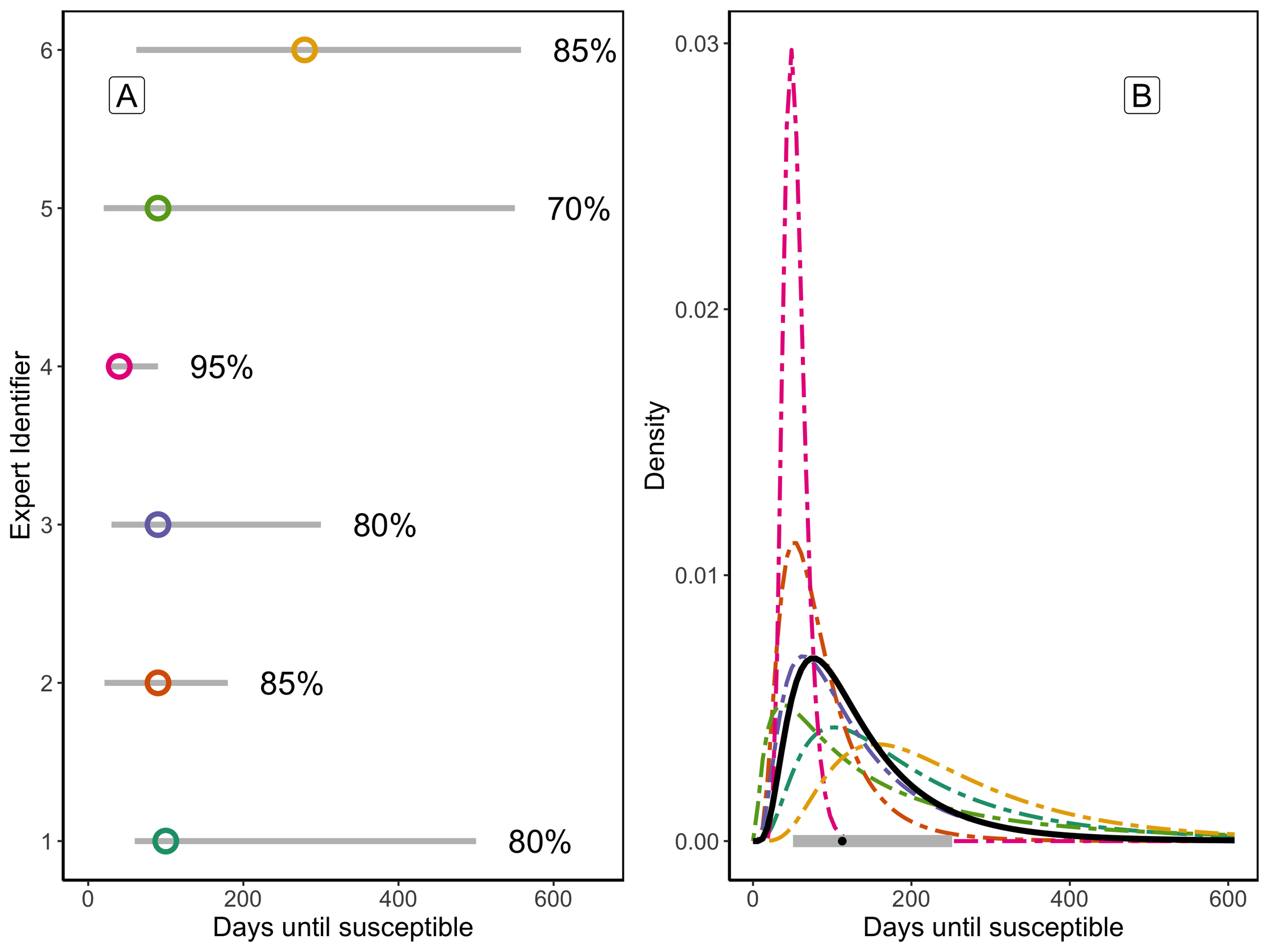

Supplement: S1 Fig — Consider a healthy individual white-tailed deer that was recently infected with SARS-CoV-2 and has since recovered (i.e., the individual is no longer shedding infectious virions). Assume that, with recovery, this individual is temporarily immune to reinfection by SARS-CoV-2 if they were to be exposed to the virus by a dose otherwise sufficient to cause infection. However, after some period, this individual will lose their immunity and become susceptible to infection again. After how many days can this individual deer be reinfected with SARS-CoV-2 after it has fully recovered from an infection? (A) fitted log-normal probability distributions for answers provided by individual experts, and (B) the aggregated log-normal distribution of answers across experts (black line). The aggregate log-normal distribution has a median of 112.6 days (80% confidence interval: 50.5–251.4 days; grey range along x-axis). (TIF) [file pcbi.1012263.s003.tif]

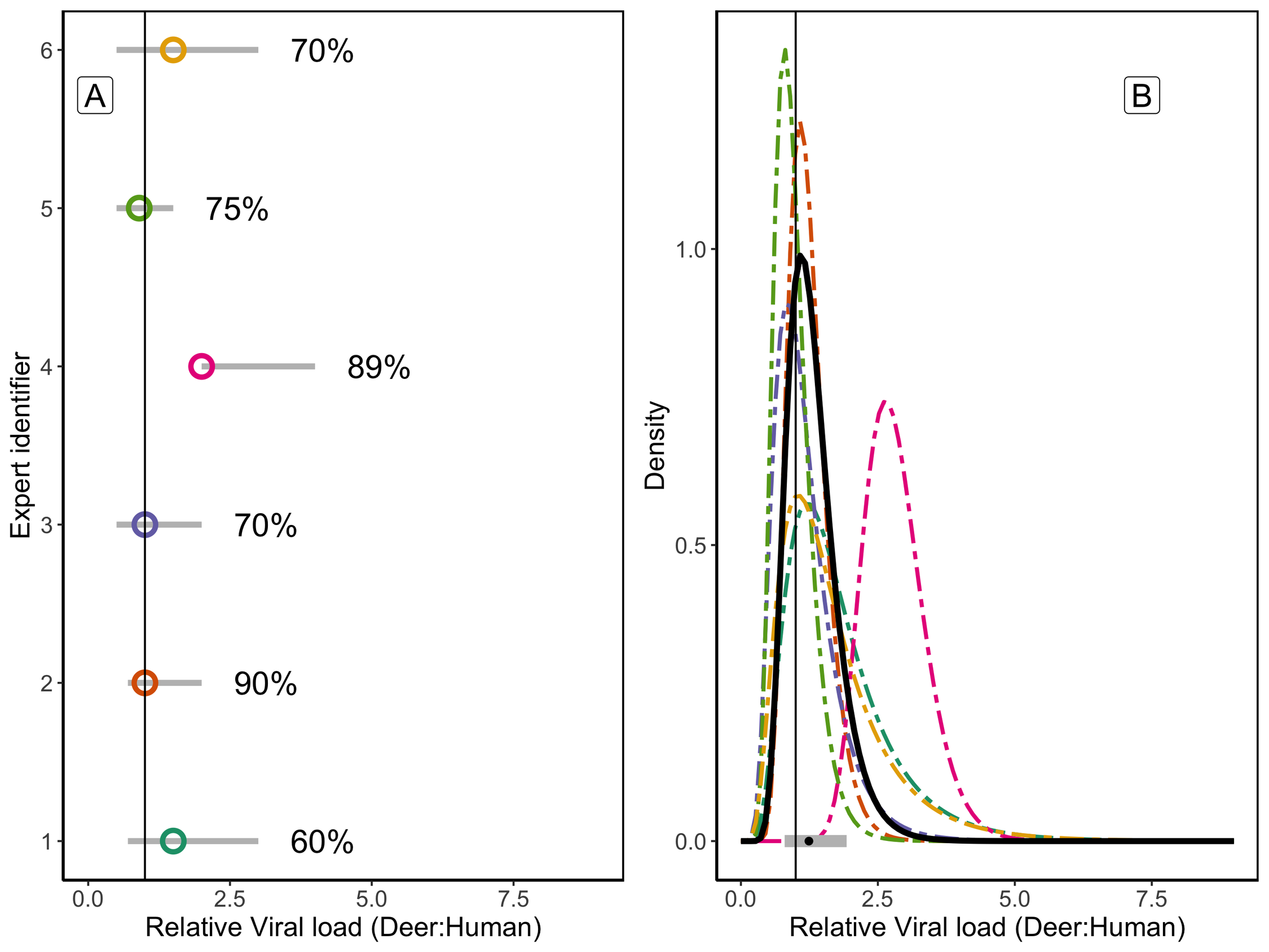

Supplement: S2 Fig — Consider a white-tailed deer infected with SARS-CoV-2 from which you can collect a sputum sample. What is the ratio of the average viral load of a deer compared to the average viral load in an infected human’s sputum sample? (A) fitted log-normal probability distributions for answers from individual experts, and (B) the aggregated log-normal distribution of answers across experts. The aggregate log-normal distribution has a median viral load of 1.24 that found in humans (80% confidence interval: 0.80–1.93; grey range along x-axis). The vertical lines in both (A) and (B) refer to a ratio of 1, that corresponds to no difference between viral loads in deer and human sputum. (TIF) [file pcbi.1012263.s004.tif]

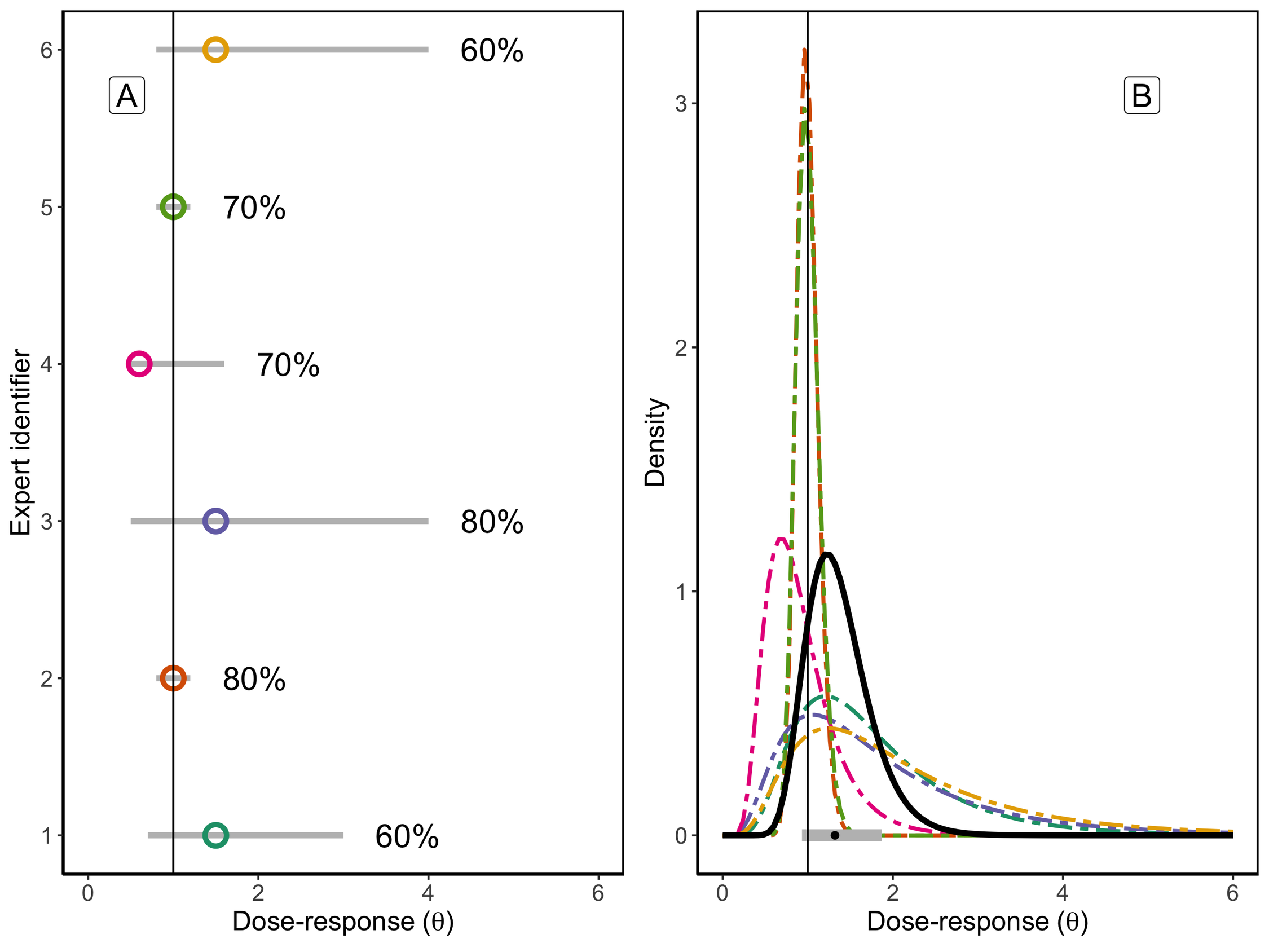

Supplement: S3 Fig — For humans, θ = 1, corresponding to a 1 quantum dose successfully infecting 63% of susceptible individuals, or HID63. Based on your expertise and knowledge of the literature, what do you expect the r value to be for the average, healthy white-tailed deer? (A) fitted log-normal probability distributions for answers from individual experts, and (B) the aggregated log-normal distribution of answers across experts. The aggregate log-normal distribution has a median dose-relationship of 1.32 (80% confidence interval: 0.93–1.87; grey range along x-axis). The vertical line indicates the human dose-response relationship, θ = 1. (TIF) [file pcbi.1012263.s005.tif]

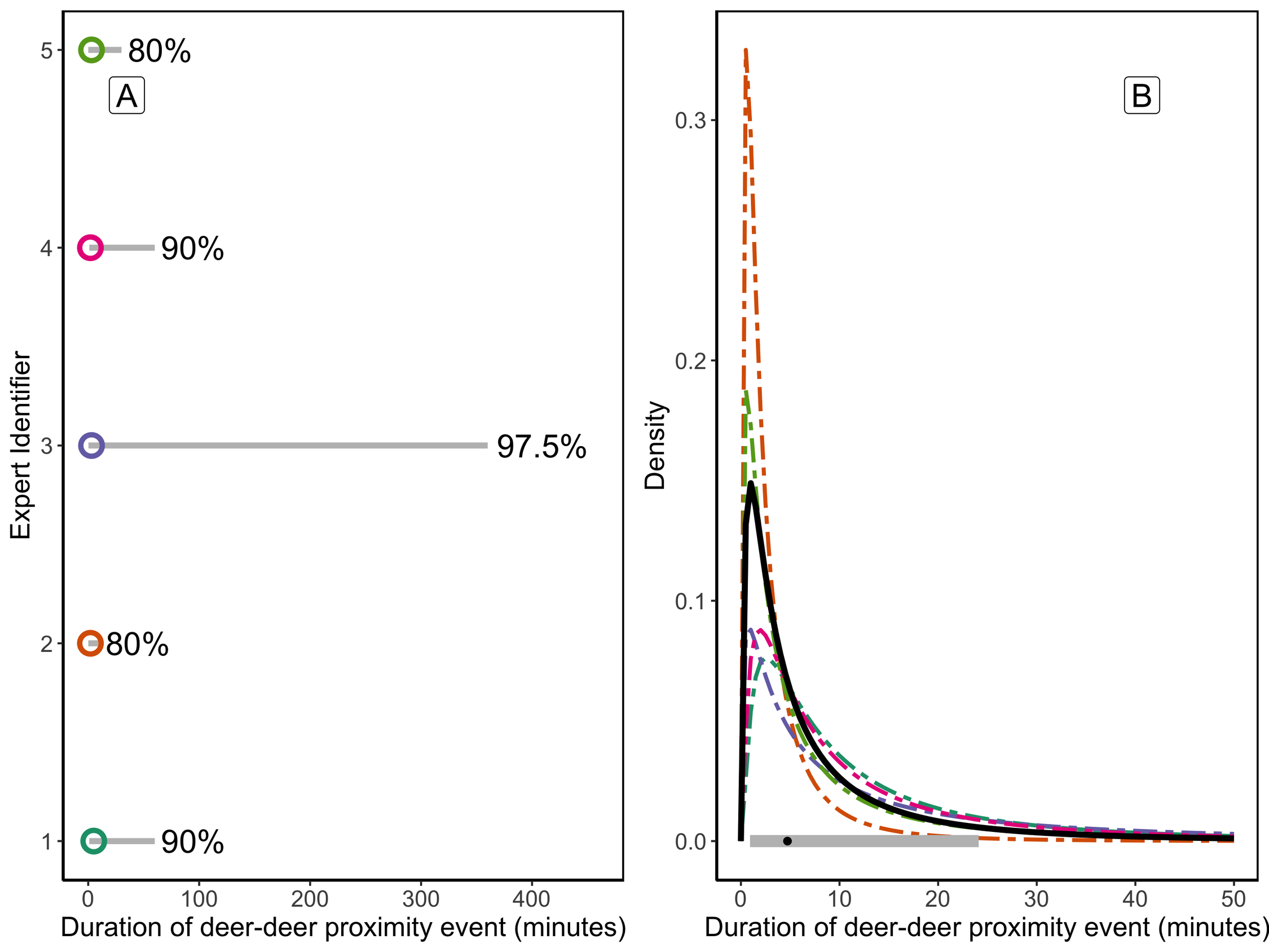

Supplement: S4 Fig — (A) fitted log-normal probability distributions for answers from individual experts, and (B) the aggregated log-normal distribution of answers across experts. The aggregate log-normal distribution has a median duration of 4.72 minutes (80% confidence interval: 0.93–24.11 minutes; grey range along x-axis). (TIF) [file pcbi.1012263.s006.tif]

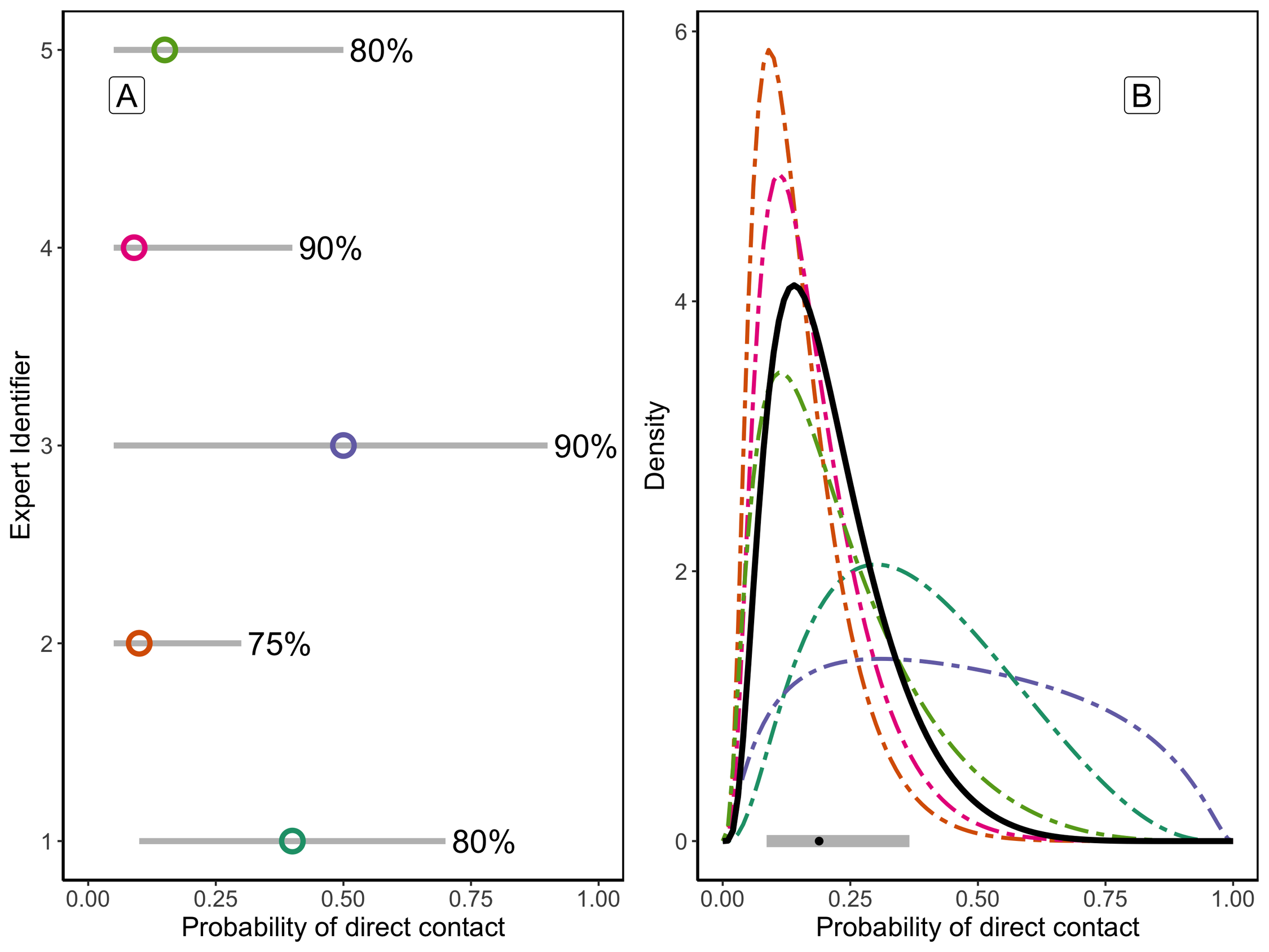

Supplement: S5 Fig — Given that two deer are in proximity (within 1.5m of each other), what is the probability that these individuals make direct contact? (A) fitted logit-normal probability distributions for answers from individual experts, and (B) the aggregated logit-normal distribution of answers across experts. The aggregate logit-normal distribution has a median direct contact probability of 0.19 (80% confidence interval: 0.09–0.37; grey range along x-axis). (TIF) [file pcbi.1012263.s007.tif]

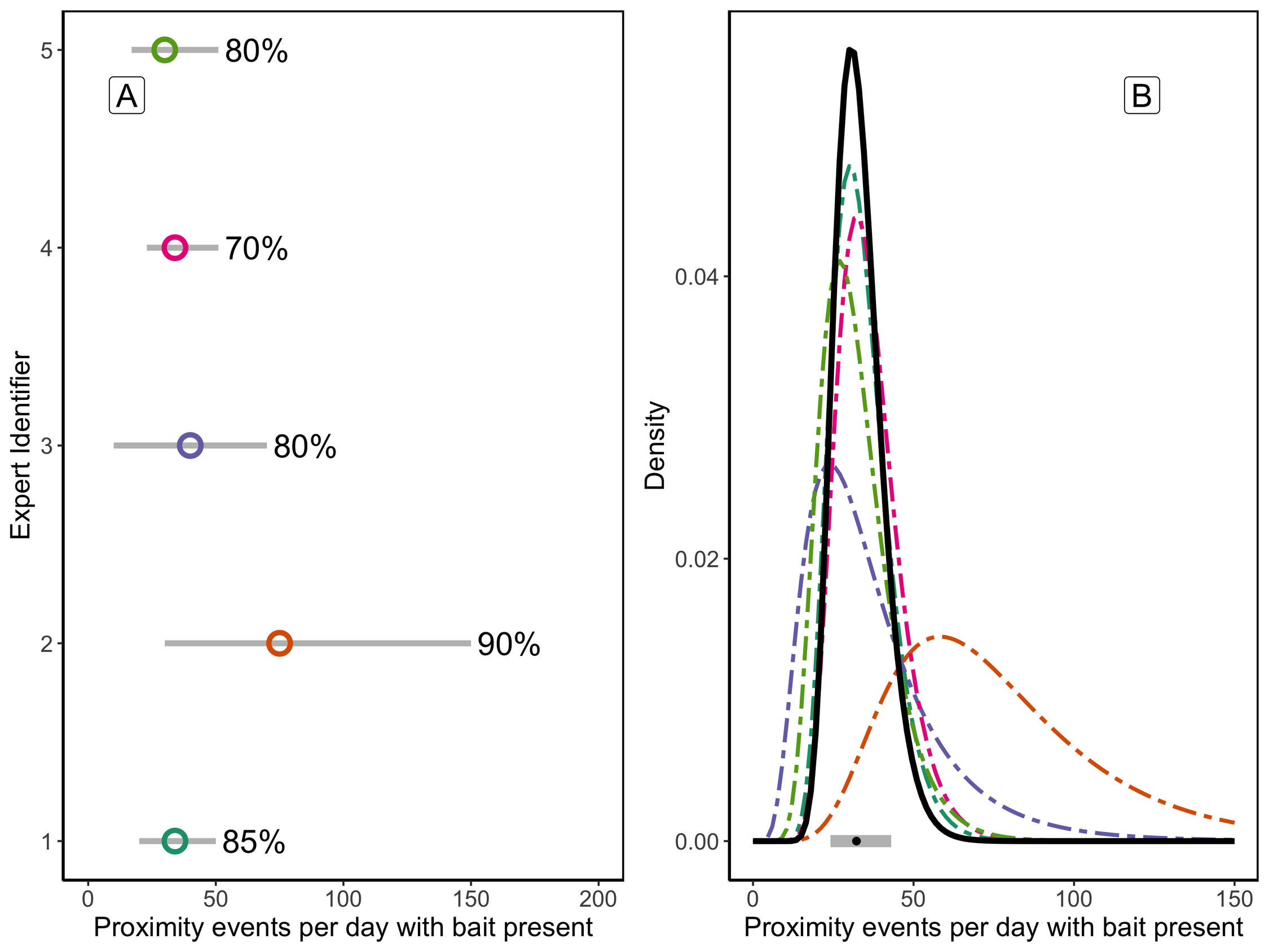

Supplement: S6 Fig — If an individual deer has 17 proximity events with other deer each day in the absence of baiting, how many proximity events do you expect an individual deer to have with other wild deer in the presence of an attractant (bait, food, or other product intended to attract deer)? (A) fitted log-normal probability distributions for answers from individual experts, and (B) the aggregated log-normal distribution of answers across experts. The aggregate log-normal distribution has a median 32.2 proximity events per day when an attractant is present (80% confidence interval: 24.1–43.0). Relative to 17 proximity events in the absence of baiting, this aggregate distribution estimates an increase in proximity events by 1.90-fold when an attractant is present (80% confidence interval: 1.42–2.53; grey range along x-axis). (TIF) [file pcbi.1012263.s008.tif]

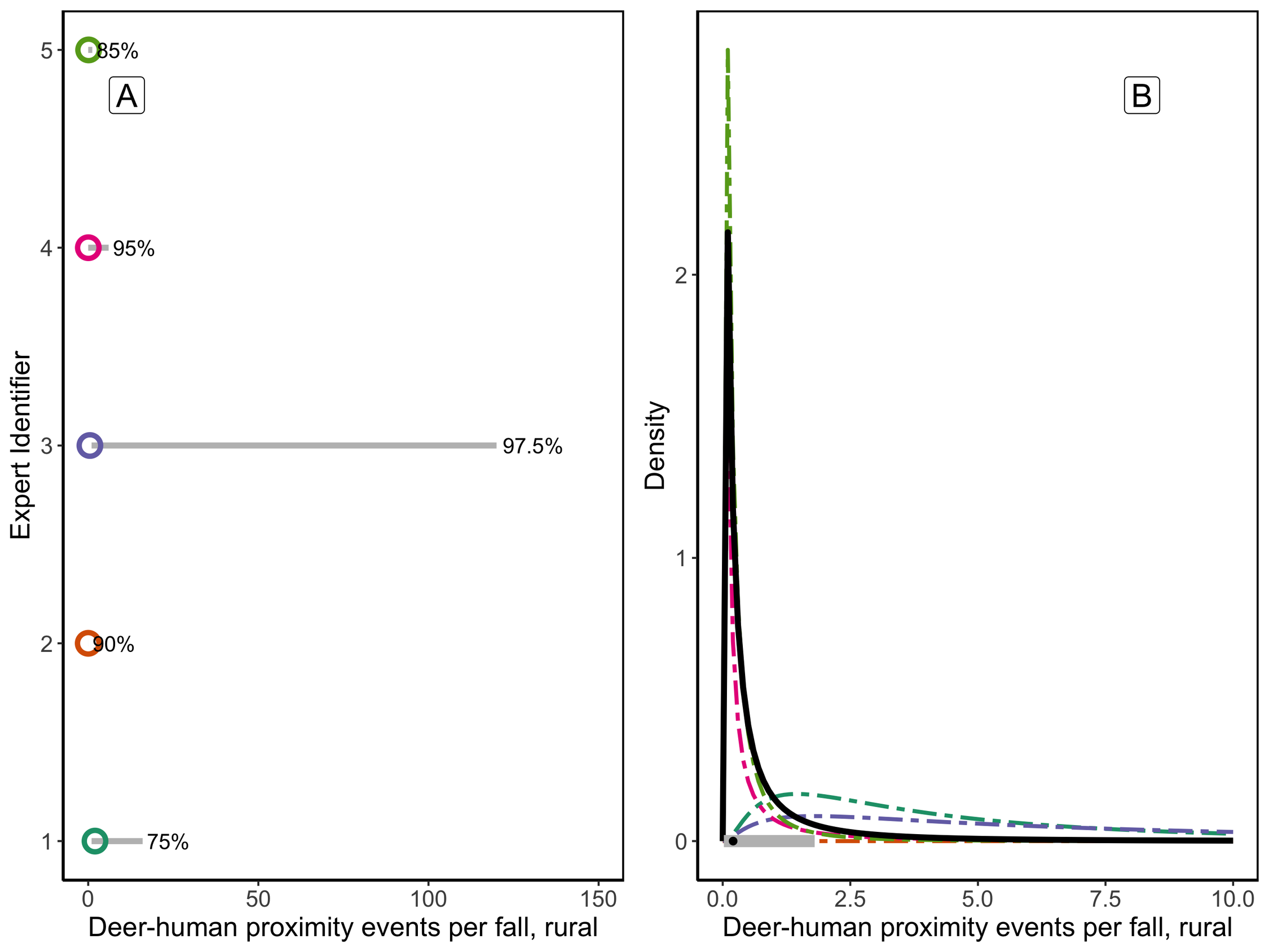

Supplement: S7 Fig — Given the conditions outlined above [S2 File], how many times do you expect an individual deer to come into proximity with a human during the fall months (1 September– 31 December)? (A) fitted log-normal probability distributions for answers from individual experts, and (B) the aggregated log-normal distribution of answers across experts. The aggregate log-normal distribution has a median of 0.20 proximity events per deer, per fall in a rural setting (80% confidence interval: 0.02–1.80; grey range along x-axis). (TIF) [file pcbi.1012263.s009.tif]

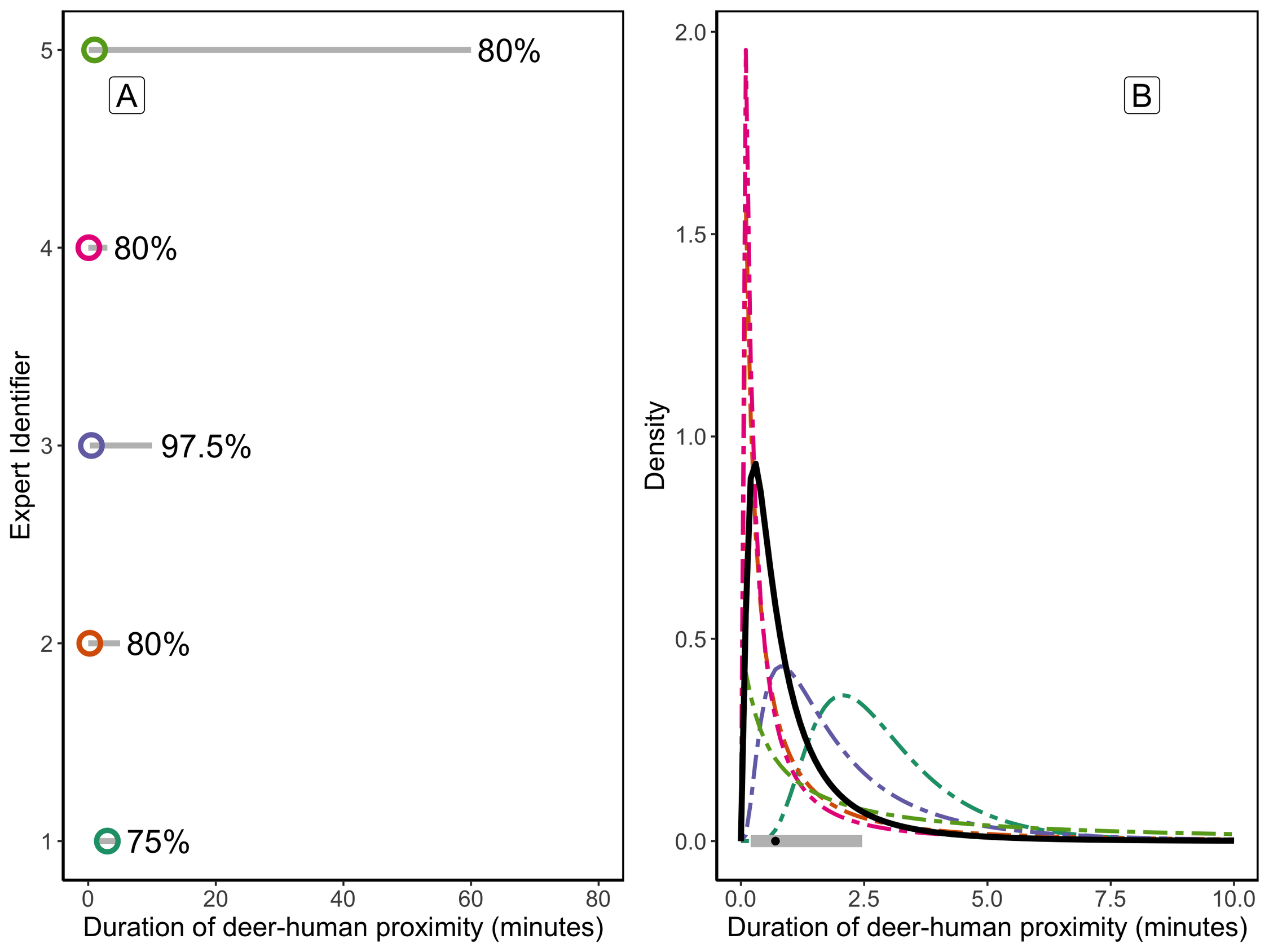

Supplement: S8 Fig — Given that a human and a deer come into proximity in a rural setting (within 1.5m of each other), how long do you expect these individuals (human and deer) to stay in proximity on average (minutes)? (A) fitted log-normal probability distributions for answers from individual experts, and (B) the aggregated log-normal distribution of answers across experts. The aggregate log-normal distribution has a median proximity duration of 0.70 minutes in a rural setting (80% confidence interval: 0.20–2.46 minutes; grey range along x-axis). (TIF) [file pcbi.1012263.s010.tif]

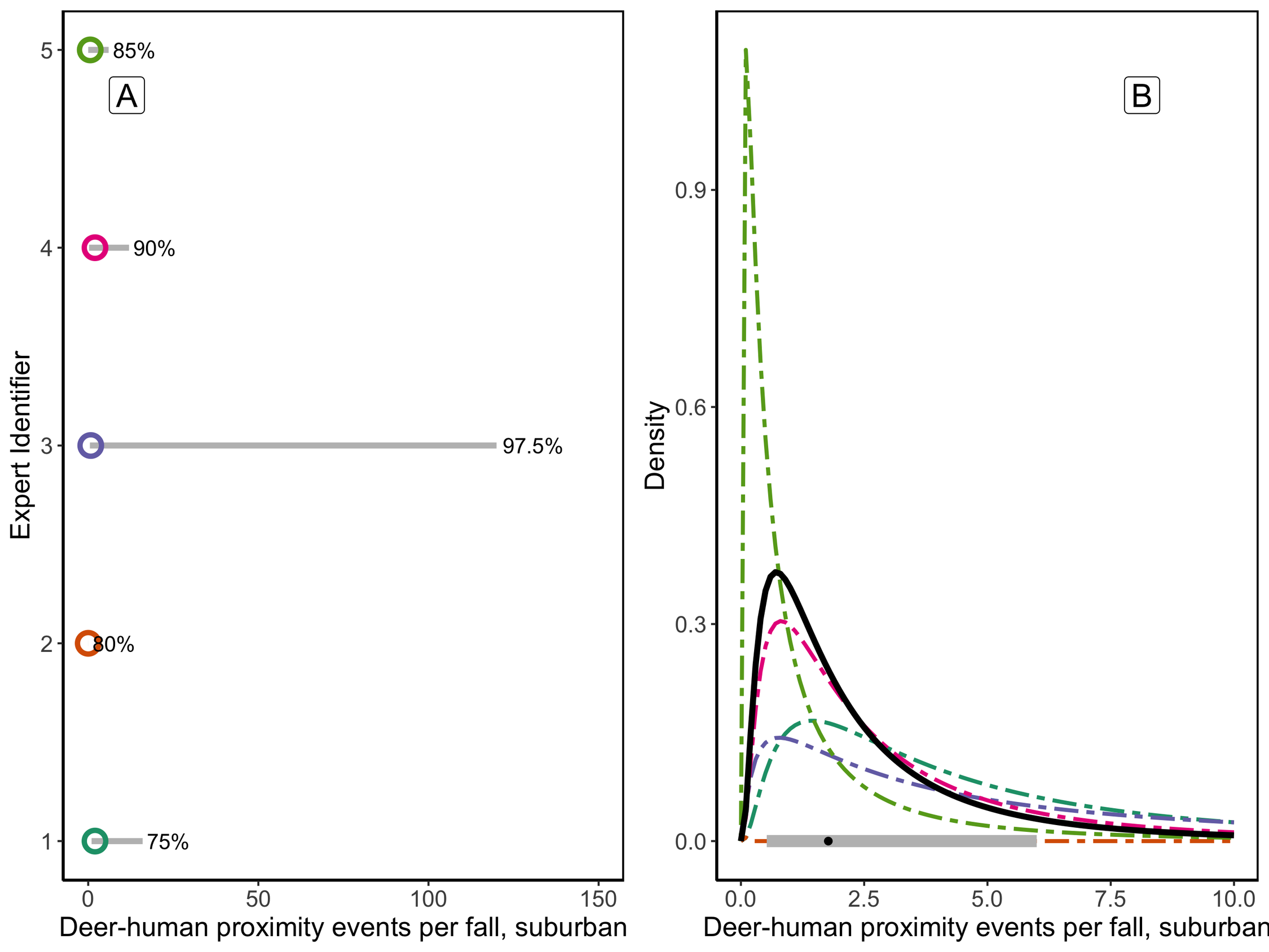

Supplement: S9 Fig — Given the conditions outlined above [S2 File], how many times do you expect an individual deer to come into proximity with a human during the fall months (1 September– 31 December)? (A) fitted log-normal probability distributions for answers from individual experts, and (B) the aggregated log-normal distribution of answers across experts. The aggregate log-normal distribution has a median of 1.77 proximity events per deer, per fall in a suburban setting (80% confidence interval: 0.52–6.00; grey range along x-axis). (TIF) [file pcbi.1012263.s011.tif]

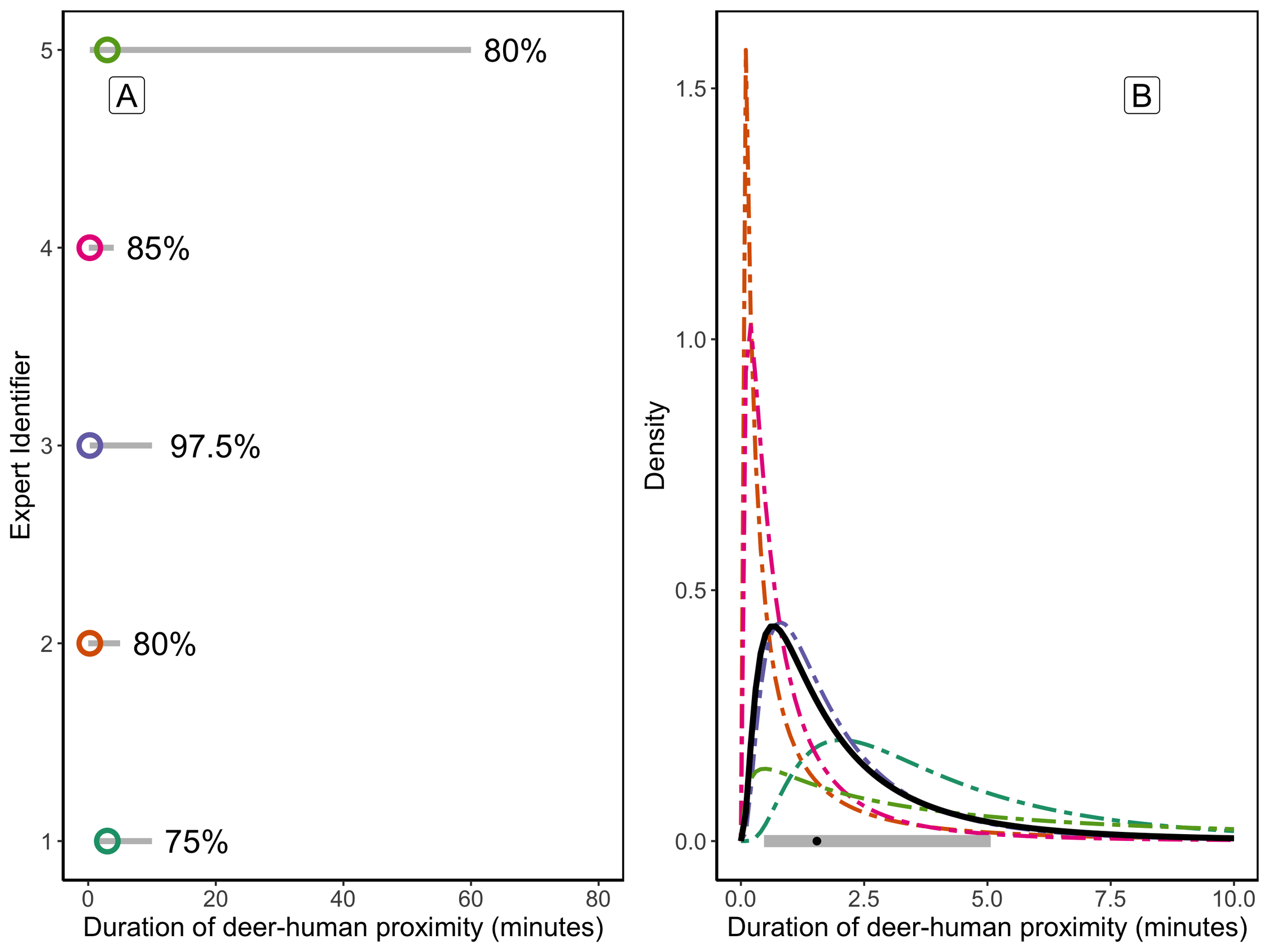

Supplement: S10 Fig — Given that a human and a deer come into proximity in a suburban setting (within 1.5m of each other), how long do you expect these individuals (human and deer) to stay in proximity on average (minutes)? (A) fitted log-normal probability distributions for answers from individual experts, and (B) the aggregated log-normal distribution of answers across experts. The aggregate log-normal distribution has a median proximity duration of 1.54 minutes in a suburban setting (80% confidence interval: 0.47–5.07 minutes; grey range along x-axis). (TIF) [file pcbi.1012263.s012.tif]

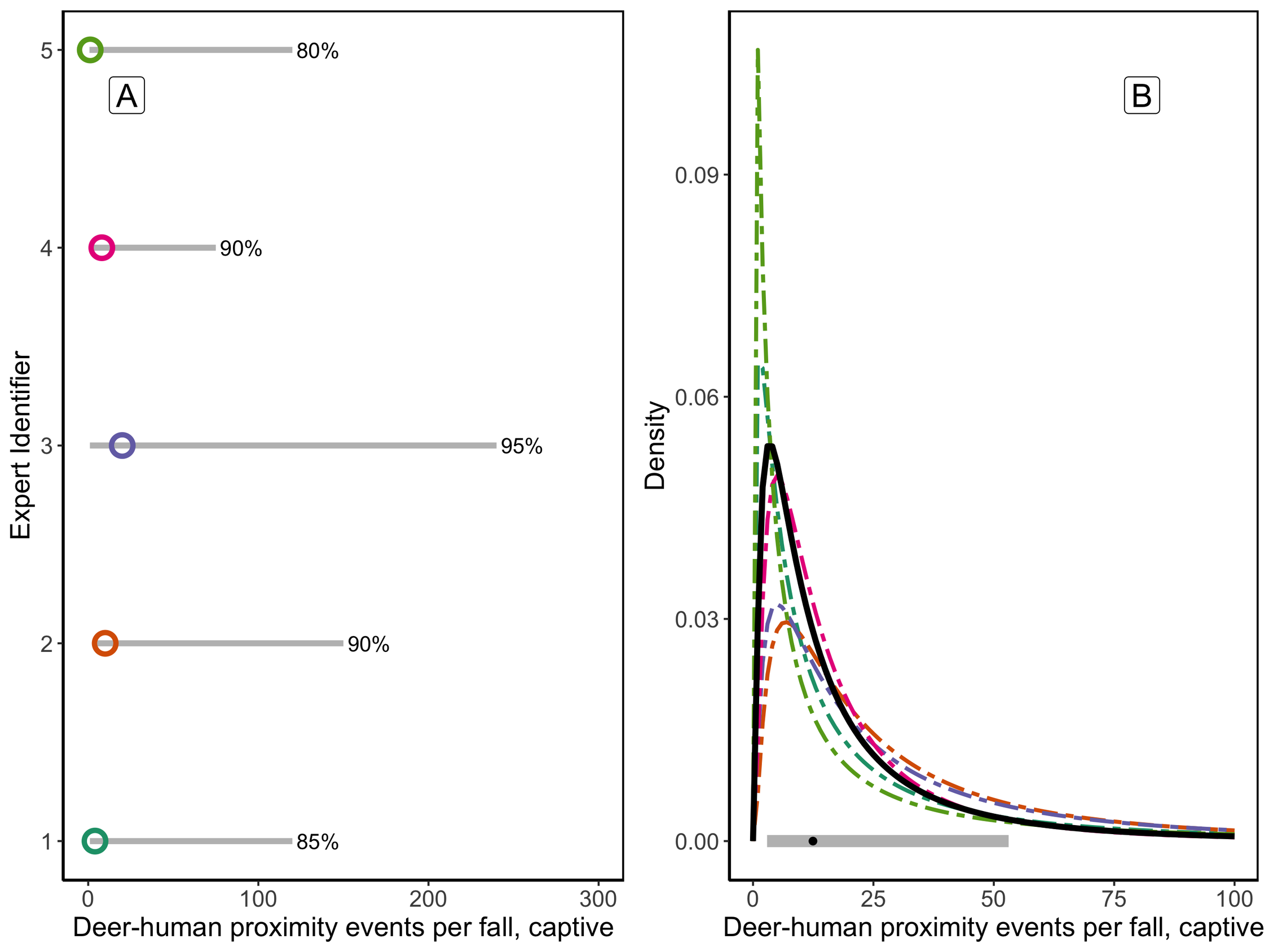

Supplement: S11 Fig — How many times do you expect an individual deer in a captive facility to come into proximity with a human (within 1.5m of each other) during the fall months (1 September– 31 December)? (A) fitted log-normal probability distributions for answers from individual experts, and (B) the aggregated log-normal distribution of answers across experts. The aggregate log-normal distribution has a median of 12.44 proximity events per deer, per fall in an intensive captive setting (80% confidence interval: 2.92–53.08; grey range along x-axis). (TIF) [file pcbi.1012263.s013.tif]

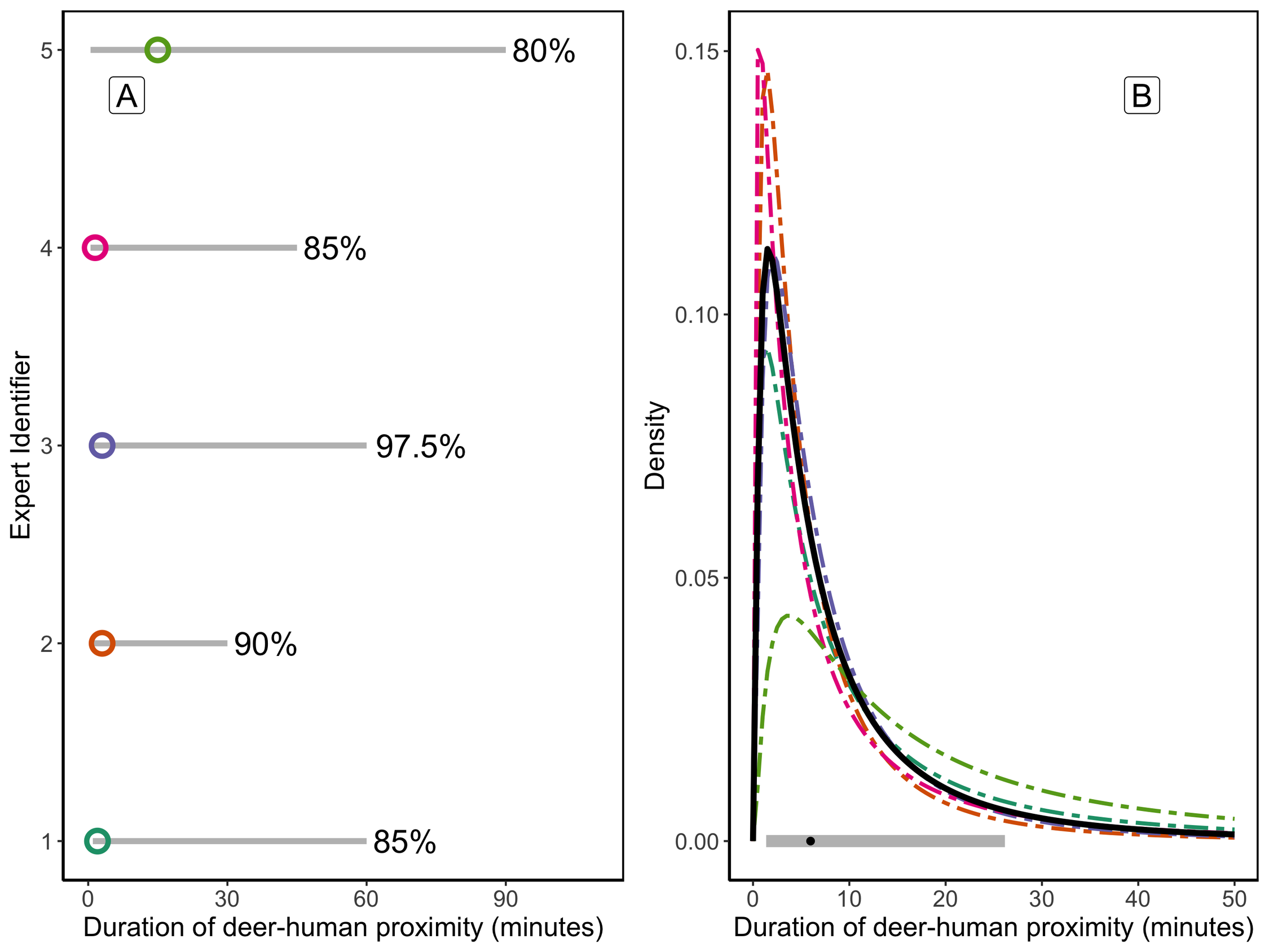

Supplement: S12 Fig — Given that a human and a deer come into proximity in a captive setting (within 1.5m of each other), how long do you expect these individuals (human and deer) to stay in proximity on average (minutes)? (A) fitted log-normal probability distributions for answers from individual experts, and (B) the aggregated log-normal distribution of answers across experts. The aggregate log-normal distribution has a median proximity duration of 5.98 minutes in an intensive captive setting (80% confidence interval: 1.36–26.16 minutes; grey range along x-axis). (TIF) [file pcbi.1012263.s014.tif]

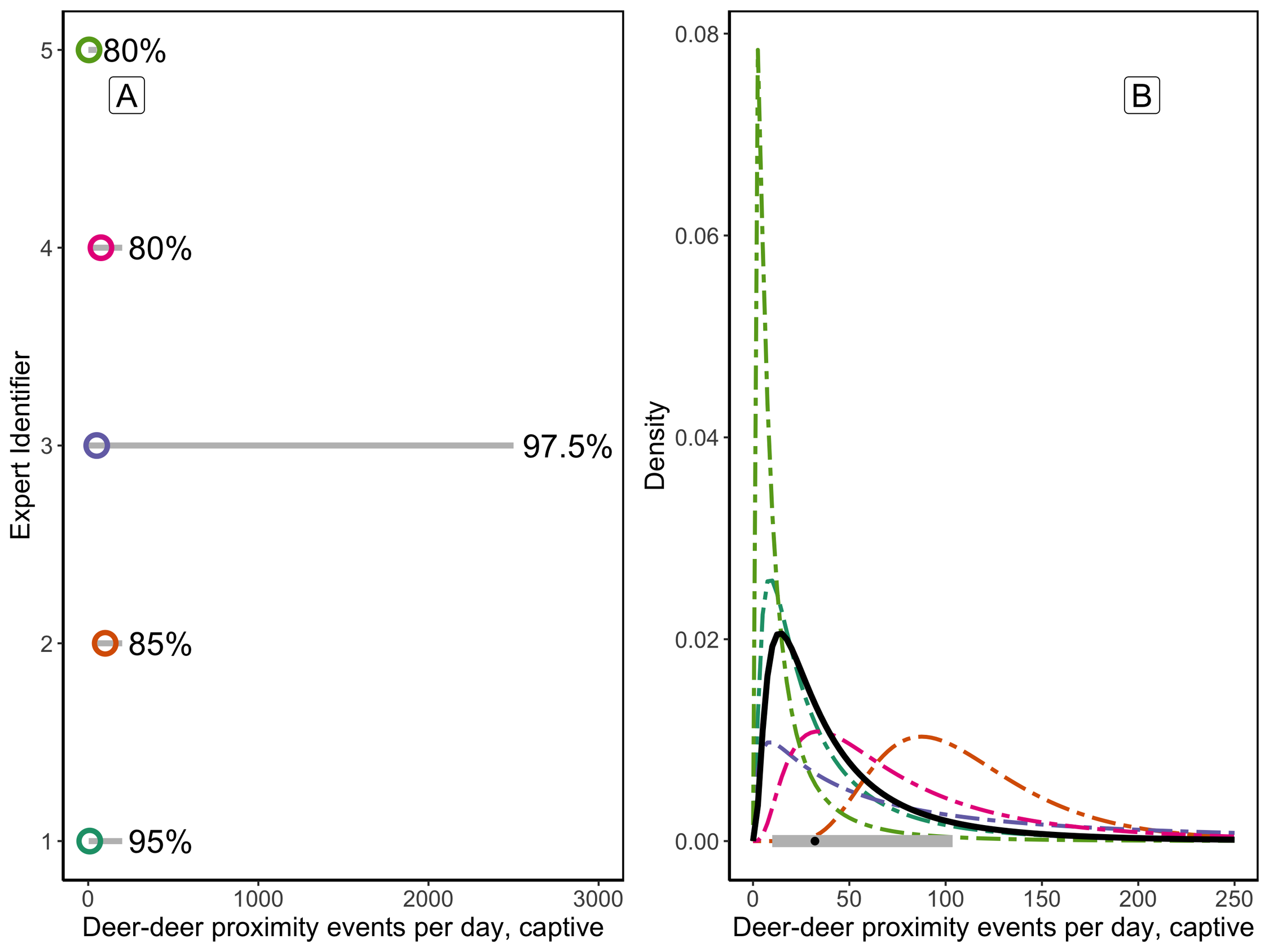

Supplement: S13 Fig — Given these captive conditions, how many times do you expect an individual deer to be in proximity with another deer in a day on average (within 1.5m of each other)? (A) fitted log-normal probability distributions for answers from individual experts, and (B) the aggregated log-normal distribution of answers across experts. The aggregate log-normal distribution has a median proximity rate of 32.15 events per day in an intensive captive setting (80% confidence interval: 9.97–103.61 events per day; grey range along x-axis). (TIF) [file pcbi.1012263.s015.tif]

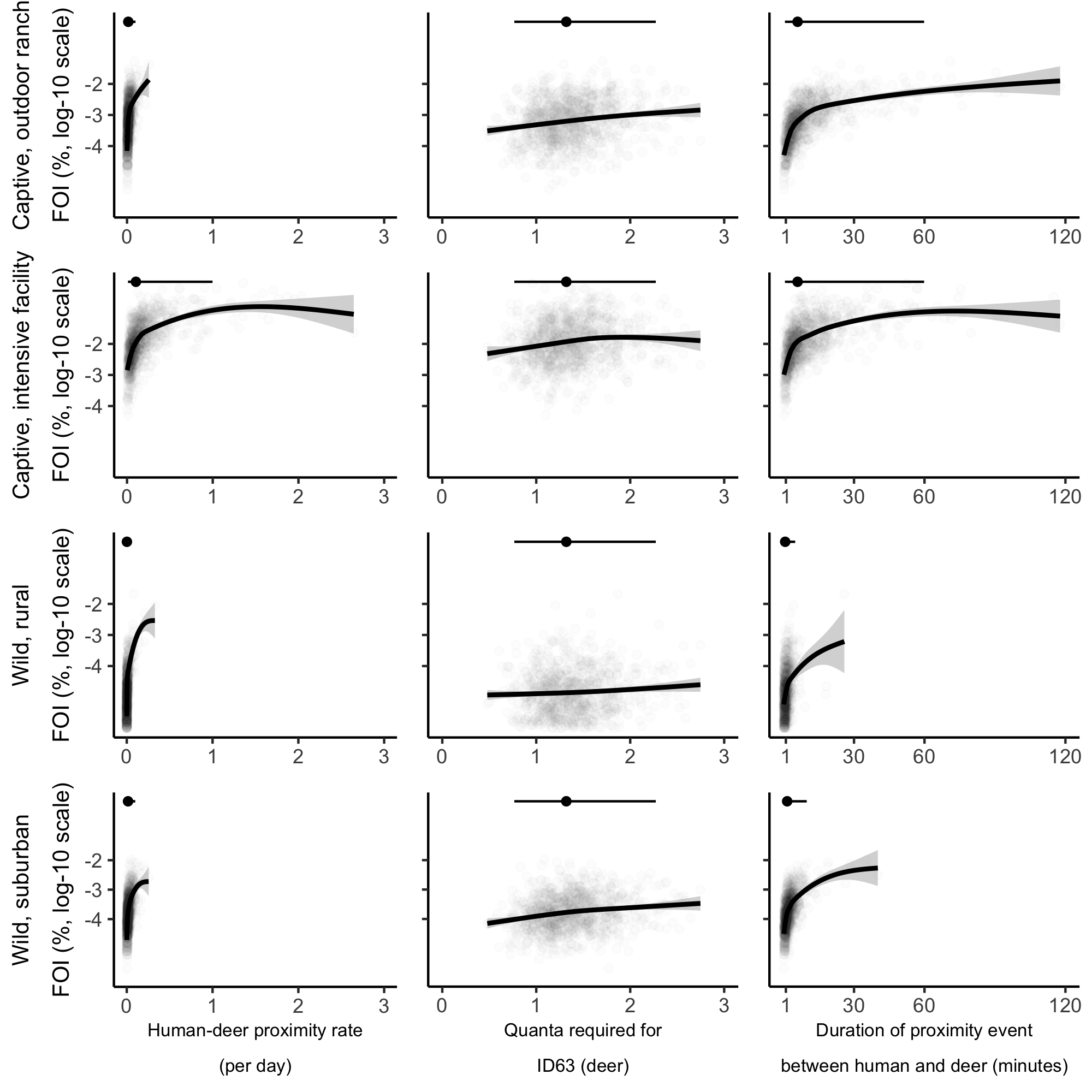

Supplement: S14 Fig — Each row corresponds to a scenario, and each column corresponds to a parameter included in the calculation of FOI. Points indicate each iteration’s draw of each parameter and resulting derived parameter (FOI), with a trend line fitted to summarize the sensitivity of FOI to the range of drawn parameter values. Point and error bars on the top of each plot indicate the mean and 95% confidence intervals of the aggregate parameter distribution from expert elicitation. (TIF) [file pcbi.1012263.s016.tif]

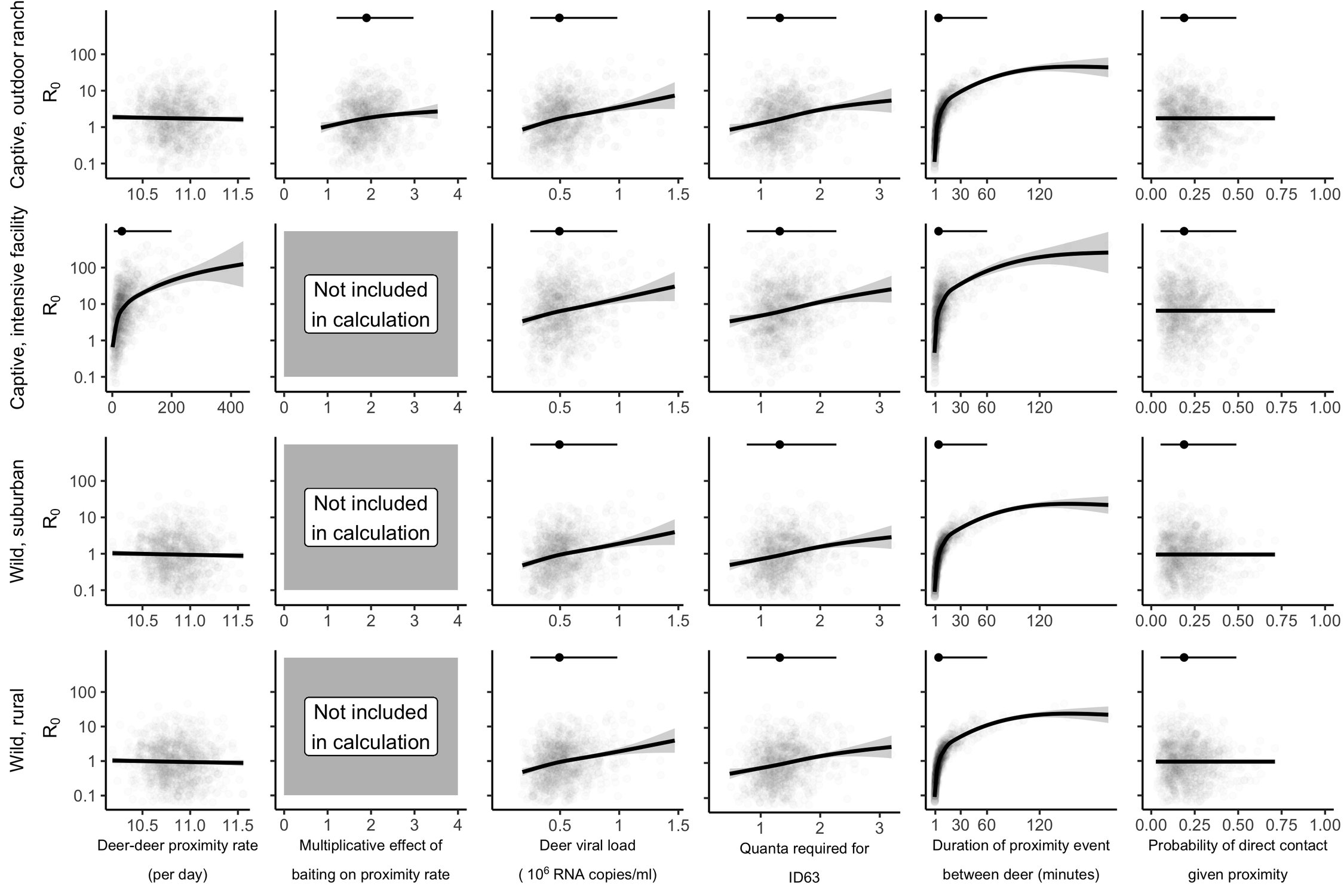

Supplement: S15 Fig — Each row corresponds to a scenario, and each column corresponds to a parameter included in the calculation of R0. Points indicate each iteration’s draw of each parameter and resulting derived parameter (R0), with a trend line fitted to summarize the sensitivity of R0 to the range of drawn parameter values. Point and error bars on the top of each plot indicate the mean and 95% confidence intervals of the aggregate parameter distribution from the expert elicitation exercise. Deer-deer proximity rates for captive, outdoor ranch, wild, suburban, and wild, rural scenarios were drawn from Habib et al.’s (2014) [32] contact rate model, with less uncertainty compared to expert-elicited proximity rates in the captive, intensive facility scenario. (TIF) [file pcbi.1012263.s017.tif]
