# Supplementary material for: Epidemiological modeling of SARS-CoV-2 in white-tailed deer (Odocoileus virginianus) reveals conditions for introduction and widespread transmission
Source: PLoS Comput Biol. 2024 Jul 12;20(7):e1012263. doi: 10.1371/journal.pcbi.1012263 (PMC11268674; doi:10.1371/journal.pcbi.1012263)
Supplement: Attachment — Submitted filename: Epi paper response to reviewers.docx [file pcbi.1012263.s018.docx]

Dear Dr. Rosenblatt,

Thank you very much for submitting your manuscript "Epidemiological modeling of SARS-CoV-2 in white-tailed deer (Odocoileus virginianus) reveals conditions for introduction and widespread transmission" for consideration at PLOS Computational Biology.

As with all papers reviewed by the journal, your manuscript was reviewed by members of the editorial board and by several independent reviewers. In light of the reviews (below this email), we would like to invite the resubmission of a significantly-revised version that takes into account the reviewers' comments.

We cannot make any decision about publication until we have seen the revised manuscript and your response to the reviewers' comments. Your revised manuscript is also likely to be sent to reviewers for further evaluation.

When you are ready to resubmit, please upload the following:

[1] A letter containing a detailed list of your responses to the review comments and a description of the changes you have made in the manuscript. Please note while forming your response, if your article is accepted, you may have the opportunity to make the peer review history publicly available. The record will include editor decision letters (with reviews) and your responses to reviewer comments. If eligible, we will contact you to opt in or out.

[2] Two versions of the revised manuscript: one with either highlights or tracked changes denoting where the text has been changed; the other a clean version (uploaded as the manuscript file).

Important additional instructions are given below your reviewer comments.

Please prepare and submit your revised manuscript within 60 days. If you anticipate any delay, please let us know the expected resubmission date by replying to this email. Please note that revised manuscripts received after the 60-day due date may require evaluation and peer review similar to newly submitted manuscripts.

Thank you again for your submission. We hope that our editorial process has been constructive so far, and we welcome your feedback at any time. Please don't hesitate to contact us if you have any questions or comments.

Sincerely,

Benjamin Althouse
Academic Editor
PLOS Computational Biology

Rob De Boer
Section Editor
PLOS Computational Biology

***********************

**Response to editor**:

Dear Dr. Althouse and Dr. De Boer,

Thank you for your response to our submission of the manuscript, “Epidemiological modeling of SARS-CoV-2 in white-tailed deer (*Odocoileus virginianus*) reveals conditions for introduction and widespread transmission.” We appreciate the constructive feedback provided by you and the two reviewers. We have carefully worked through all comments and suggestions. We have included point-by-point responses to reviewer comments below and have attached an updated draft with track changes to highlight the changes. We felt it was important to highlight the major improvements to the manuscript here as well, primarily:

- The addition of supplemental plots displaying sensitivity of basic reproductive number (R_0_) and Force-Of-Infection (FOI) to expert-elicited parameters, as suggested by Reviewer 1;
- The reframing of Objective 3 as a sensitivity analysis, following suggestions by Reviewer 1;
- Further clarity in methods, especially around discrete versus continuous interpretation of SIRS ordinary differential equations (ODE), as suggested by Reviewer 1.

Thanks to one reviewer’s suggested sensitivity analysis, we found that our code projecting SARS-CoV-2 outbreaks used an incorrect proximity rate between humans and deer. With this correction, our results suggested even higher spillover risk and outbreak metrics in our simulated captive deer population in an intensive facility. We have incorporated these changes in the manuscript, though our broader inferences remained the same. We have double-checked the simulation code for any other incorrect parameter calls and found no other issues.

We found the reviewers’ comments constructive, and they have helped to make the manuscript more accessible to a broader audience and the inferences clearer.

For all authors,

Dr. Elias Rosenblatt

**Response to reviewers’ comments:**

Author responses are in red, with quoted passages in italics and any alterations underlined.

Editorial changes:

Table 1: Deleted last parameter (C_v-deer_) as it is listed earlier in the table.

We discovered a coding error for proximity rate in the intensive captive scenario, where previous results were reported using the same proximity rate as captive ranch and wild, suburban scenarios. The corrected code results in higher rates of human-to-deer spillover of SARS-CoV-2 in the intensive captive deer scenario. The results, Tables 2 and 3, and Figures 3, 4, 5, and 6 have been updated. Our inferences and discussion did not change. Results section changes include:

Line 523-525: Originally read: “*Median FOI_HD_ estimates were 219-, 85-, and 19-times higher in the intensive facility, outdoor ranch, and wild deer in suburban scenarios, respectively, relative to median FOI_HD_ estimates for rural, wild deer (Table 2).*” This passage now reads: “*Median FOI_HD_ estimates were 1244-, 85-, and 19-times higher in the intensive facility, outdoor ranch, and wild deer in suburban scenarios, respectively, relative to median FOI_HD_ estimates for rural, wild deer (Table 2).*”

Line 526-528: Originally read: “*Median probabilities of at least one HtD transmission per 1000 deer ranged from 88%, 56.1%, 17.7%, and 1.1% in the intensive facility, outdoor ranch, wild suburban, and wild rural scenarios, respectively (Table 2).*” This passage now reads: “*Median probabilities of at least one HtD transmission per 1000 deer ranged from 100%, 56.1%, 17.7%, and 1.1% in the intensive facility, outdoor ranch, wild suburban, and wild rural scenarios, respectively (Table 2).*”

Reviewer #1: This manuscript is an interesting exploration of the spillover dynamics of SARS COVID19 from humans into white-tailed deer. I thought the expert elicitation exercise was an interesting way to obtain estimates of rates that are needed to parameterize multiple routes of spillover.
I am skeptical that the expert elicitation combined with literature defined rates of fine-scale infectivity and viral parameters from model systems is able to make the pathways identifiable. That said, I think the conclusions that are centered on that results that it takes a very little amount of spillover from humans can seed transmission are valuable.

We appreciate the reviewer’s perspective on the use of estimates from existing literature and expert elicitation. We highlight that for any assessment to be made on this topic, we needed to fill critical gaps in the current published literature using alternative means. While empirical data are ideal, they do not exist for this system. Instead, we used established best practices for elicitation of expert judgment using a 4-point elicitation method, which incorporates expert uncertainty in our parameter estimates, and integrated these estimates (including uncertainty) into our SIRS framework. Expert judgment methods, when conducted with the same level of rigor as the collection and use of empirical data, can result in reliable predictions (for example, O’Hagan and others, 2006; Speirs-Bridge and others, 2010; Runge and others, 2011; Martin and others, 2012; Adams-Hosking and others, 2016). We also highlight that our expert panels were comprised of well-qualified scientists who are active in research topics related to the parameters in question.

Using these estimates, we detected differences in Force-of-Infection (FOI) and probability of a human-deer spillover event (p(HtD, 1:1000)) between multiple scenarios (Table 2). The only derived parameter driving FOI and p(HtD, 1:1000) was human-to-deer transmission rate, which was informed by expert-elicited parameter values. Despite the uncertainty nested in parameter values that lead to these transmission rates, we can still identify differences in the resulting spillover hazard. Ideally, we could garner more precise estimates for all pathways considered in this manuscript, but this improvement in precision will only be gained through further study. We agree with the reviewer, that uncertainty in parameter estimates used in this study do not undermine the primary conclusions presented.

Sensitivity and expert elicitation:
I think that the exploration of parameter space was sufficiently justified via the expert elicitation and scenario set-up. However, there is also little preventing more thorough exploration of the sensitivity of the objective outputs in broader parameter space. Particularly, Objective 1: In reading the methods, my initial thought was that this would be an ideal place to perform more thorough sensitivity analyses on the effect of the parameter uncertainty because its an analytical solution. Then line 510 in results describes variation in the FOI calculations. Was this a sensitivity analysis? Or incorporated uncertainty from the expert elicitation? How that variation in the FOI solutions needs to be included in the methods.

The reviewer brings up an important point that strengthens this manuscript. In simulation, we better explored the parameter space by taking random draws from probability distributions for all parameters (where distributions were derived for all parameters from a combination of literature and expert-elicited estimates) and used those random draws to project outbreak dynamics in simulated deer populations [see Lines 456-461]. Originally, we did not conduct a sensitivity analysis of spillover (FOI) and spread (R_0_) using the various expert-elicited parameters. We have added language detailing a sensitivity analysis for Objective 1 and changed language for Objective 3 to highlight our investigation of the sensitivity of various outbreak metrics to introduction and spread. These changes include:

Objective 1:

We added a sensitivity analysis to this objective and focused on presenting the sensitivity of FOI and R_0_ to expert-elicited parameters, as suggested by the reviewer. Given the focus of the manuscript and the number of figures already presented in the main body of the manuscript, we chose to report these additional figures in the Supplemental Materials.

In the methods for Objective 1, we added the following passage (Lines 450-454): “*We summarized the sensitivity of FOI_HD_ and* R_0_ *to expert-elicited parameters in the supplemental materials. We focused on expert-elicited parameters for these sensitivities as these parameters had the greatest uncertainty in our calculations. We did not present sensitivity of p(HtD) to expert-elicited parameters as p(HtD) was derived from FOI.”*

In Objective 1’s results section, we added the following two passages:

Lines 525-526: “*FOI_HD_ was highly sensitive to the frequency and duration of proximity between humans and deer (S14).”*

Lines 538-542: *“*R_0_ *was sensitive to several parameters, including duration of a deer-deer proximity event, the concentration of SARS-CoV-2 in deer sputum, and SARS-CoV-2 dose-response in deer (S15).* R_0_ *in captive, intensive facilities was sensitive to deer-deer proximity rate due to the uncertainty around the aggregate estimate from expert elicitation (S15).”*

We added two supplemental figures (S14 Figure and S15 Figure), with the following figure captions:

S14 Figure (Lines 1073 – 1078): *“Sensitivity of Force-Of-Infection (FOI) to expert-elicited parameters. Each row corresponds to a scenario, and each column corresponds to a parameter included in the calculation of FOI. Points indicate each iteration’s draw of each parameter and resulting derived parameter (FOI), with a trend line fitted to summarize the sensitivity of FOI to the range of drawn parameter values. Point and error bars on the top of each plot indicate the mean and 95% confidence intervals of the aggregate parameter distribution from expert elicitation.”*

S15 Figure (Lines 1080 – 1088): *“Sensitivity of basic reproductive number (R0) to expert-elicited parameters. Each row corresponds to a scenario, and each column corresponds to a parameter included in the calculation of R0. Points indicate each iteration’s draw of each parameter and resulting derived parameter (R0), with a trend line fitted to summarize the sensitivity of R0 to the range of drawn parameter values. Point and error bars on the top of each plot indicate the mean and 95% confidence intervals of the aggregate parameter distribution from the expert elicitation exercise. Deer-deer proximity rates for captive, outdoor ranch, wild, suburban, and wild, rural scenarios were drawn from Habib et al.’s (2014) contact rate model, with less uncertainty compared to expert-elicited proximity rates in the captive, intensive facility scenario.”*

Objective 3:

We explored the sensitivity of outbreak metrics to FOI under different spread conditions (quantified by R_0_) in Objective 3 of the results (see Figure 5) to assess the robustness of our inference.

To highlight our existing efforts to assess the effect of uncertainty on our results, we’ve changed language around Objective 3 to explicitly center the sensitivity analysis in this objective. We’ve changed Objective 3’s listing in the introduction [Lines 134-136] from “*Understand the interaction among introduction, transmission, prevalence, persistence, and incidence proportion across all scenarios*;” to “*Understand the sensitivity of prevalence, persistence, and incidence proportion to introduction and spread across all scenarios*;

We changed Objective 3’s methods from [Lines 493-501]: “*Objective 3: Interaction between spillover, spread, prevalence and persistence – After each iteration, we categorized outcomes by one of the following spread categories: unsustained spread (R0 <1), low, sustained spread (1< R0 < 3), medium, sustained spread (3 < R0 < 5), and high, sustained spread (R0 > 5). We used the stat-smooth() function from the ggplot2 package [54] to visualize trends between HtD transmission, as quantified by FOI, prevalence, and persistence of SARS-CoV-2 for each spread category*.”

The methods passage now reads: *“Objective 3: Sensitivity of prevalence, persistence and incidence proportion to spillover and spread – We tested the sensitivity of prevalence, persistence, and incidence proportion of SARS-CoV-2 in white-tailed deer to different levels of spillover (FOI) and spread (R_0_). After each iteration, we categorized outcomes by one of the following spread categories: unsustained spread (R_0_ <1), low, sustained spread (1< R_0_ ≤ 3), medium, sustained spread (3 < R_0_ ≤ 5), and high, sustained spread (R_0_ > 5). We used the stat-smooth() function from the ggplot2 package [54] to visualize trends between HtD transmission, as quantified by FOI, and outbreak metrics for each spread category.”*

We also altered the results section for Objective 3 to highlight the sensitivity analysis explicitly [Lines 579-593]. The passage originally read: “***Objective 3: Interaction between spillover, transmission, prevalence, incidence proportion, and persistence*** *When we partitioned the relationship between FOIHD and outbreak characteristics, we found compelling evidence that FOIHD differs depending on how quickly SARS-CoV-2 transmits (R0, Fig 5). When transmission is too low to sustain SARS-CoV-2 (R0 <1 deer infected by an infected deer), high FOIHD is required for non-zero average prevalence and incidence proportion during the projection, and for a high probability of infections persisting past the 120-day projection (Fig 5). As transmission reaches self-sustaining levels (1< R0 <3 deer infected by an infected deer), the role of FOIHD has a greater influence on average prevalence, incidence proportion, and persistence (Fig 5). As R0 continues to increase to medium (3< R0 < 5 deer infected by an infected deer) and high spread (R0 > 5 deer infected by an infected deer), the influence of FOIHD on prevalence and incidence proportion diminishes, and persistence is no longer sensitive to changes in FOIHD. (Fig 5).”*

The passage now reads: “***Objective 3: Sensitivity of prevalence, persistence and incidence proportion to spillover and spread*** *When we partitioned the relationship between FOI_HD_ and outbreak characteristics, we found evidence that sensitivity to FOI_HD_ differs depending on how quickly SARS-CoV-2 transmits (R_0_, Fig 5). When deer-deer transmission is too low to sustain SARS-CoV-2 infections (R_0_ <1), high FOI_HD_ is required for non-zero average prevalence and incidence proportion during the projection, and for a high probability of infections persisting past the 120-day projection (Fig 5). As deer-deer transmission reaches self-sustaining levels (1< R_0_ <3), the role of FOI_HD_ has a greater influence on average prevalence, persistence, and incidence proportion (Fig 5). As R_0_ continues to increase to medium (3< R_0_ < 5) and high spread (R_0_ > 5), the sensitivity of prevalence and incidence proportion to FOI_HD_ diminishes, and persistence is no longer sensitive to changes in FOI_HD_. (Fig 5).*”

Additional comments are focused on clarifications of methods and interpretations:
Line 228- Clarify what you are deriving the transmission rates from up front. It will be very helpful to give a short explanation that you are deriving the rates by explicitly parameterizing contact and infectiousness, here, because this is where you first introduce this method. I was confused by how you were deriving these until I read through the methods several times.

We agree that the reader would benefit from a brief explanation of how we derived transmission parameters earlier on in the Methods section. We’ve added the following sentence at the end of the paragraph referenced by the reviewer [Lines 238 – 241]: *“We derived transmission rates as the product of HtD and DtD proximity rates and infection probabilities from previous studies. We used expert elicitation to estimate any parameters unavailable in the literature. For more detail about parameter estimation, see the Scenario Descriptions section below.”*

Line 240 and parameter table: As I understand your methods, this integrates over continuous time so there are no daily change. I suggest cleaning up the explanation of parameter units to indicate that the rates are expressed in units that have a more natural interpretation, but are all transformed to be on the equivalent scale to be integrated over your 120 day time period

We agree with the reviewer that this note is important to readers. We replaced instances of “daily change” in our SIRS equation definitions to “change” to reflect the continuous nature of these ordinary differential equations. For example, one sentence originally read: “*Three ODEs describe the disease dynamics in the wild deer population, with the daily change in the fraction of the wild population that is susceptible (sW) given by…*”

*This sentence now reads [Lines 244 – 245]: “Three ODEs describe the disease dynamics in the wild deer population, with the change in the fraction of the wild population that is susceptible (sW) given by…”.* All following text introducing components of the SIRS ODE reflects this change.

*We also added a sentence at the end of the section introducing the SIRS ODEs to orient the reader to our interpretation of these projections. This added sentence reads [Lines 275-276]: “We note that we summarized these continuous changes into discrete, daily Susceptible, Infected, and Recovered compartment sizes for our analysis for ease of interpretation.”*

We chose not to adjust the table legend to include this information as it would be repetitive. This table reports the parameters used from other published studies and from our expert panelists, with rates from discrete time periods designated by the original investigation.

Line 392-393: Clarify this sentence. I don’t understand what the 2 proximities are in this assumption

We thank the reviewer for their suggestion for making this sentence clearer. The original sentence read: “*We assumed that Habib et al.’s [32] estimated 25m proximity rate applied to our definition of proximity of 1.5m for aerosol transmission*.”

The revised sentence now reads [Lines 397-400]: “For aerosol transmission, *we assumed that proximity rates for deer approaching within 1.5m of each other were equal to Habib et al.’s [32] estimated proximity rate of deer approaching within 25m of each other*.”

Line 520-521: The statement that the R0 values with median 0.97 were too small to sustain transmission seems like too much interpretation for the results. Particularly when the range you report in Table 2 appears to include many R0 values >>1. I suggest adding a text description of the variation in R0 values in this section, in addition to presenting the median values.

We appreciate the reviewer’s comment here and have changed the sentence to reflect a more specific representation of the results. The original sentence read: *“Transmission in both wild scenarios were nearly identical, with most iterations resulting in R_0_ values too small to sustain transmission of SARS-CoV-2 (median R_0_ = 0.97; Table 2).”*

The sentence now reads [Lines 535-537]: *“Transmission in both wild scenarios were nearly identical, with 51.3% of iterations resulting in R_0_ values too small to sustain transmission of SARS-CoV-2 (R_0_ <1; median R_0_ = 0.97; Table 2).”*

Line 560-561: remove the text “deer infected by infected deer” in the parentheses. It’s confusing and you already have defined your interpretation of R0 for readers.

We agree with the reviewer’s suggestion to remove unnecessary and distracting text from the presentation of results. This issue is repeated in this particular section, so we’ve removed this text from repeated use. The sentence referenced by the reviewer originally read: *“When transmission is too low to sustain SARS-CoV-2 (R0 <1 deer infected by an infected deer), high FOIHD is required for non-zero average prevalence and incidence proportion during the projection, and for a high probability of infections persisting past the 120-day projection (Fig 5).”*

The sentence now reads [Lines 584-587]: *“When deer-deer transmission is too low to sustain SARS-CoV-2 (R0 <1), high FOI_HD_ is required for non-zero average prevalence and incidence proportion during the projection, and for a high probability of infections persisting (Fig 5).”*

-line and page numbering stopped near the end of the results and I’ll do what I can to orient the remaining comments-

We thank the reviewer for this note. We have updated this error, and now the manuscript draft has line numbers through its entirety.

Discussion sentence beginning “Our findings indicated…” [Line 651-653]
You did not include any aspect of sociality. Some aspects were including in how you modeled transmission according to previous studies, but these were constant, as far as I could tell. Remove this conclusion because the models can’t disentangle the sociality of deer (implicit in the transmission parameterization) from any of the other rates you parameterized from literature or your expert elicitation.
I also don’t think the term environmental context correctly reflects what was the focus of the modeling scenarios. I suggest describing this as “epidemiological conditions” or “human and white-tailed deer interaction context”, or something similar to more precisely convey what varied among the scenarios.

We appreciate the reviewer’s perspective here and agree that we could be more explicit. However, social behavior is accounted for in the contact rate model used in this study, after accounting for deer density and wooded habitat availability. We feel that mentioning sociality of deer is appropriate, as that is what drives contact rates, the derived transmission rates, and outbreak dynamics. However, we agree that we should be more explicit that sociality is one of the drivers of these epidemiological conditions. We also agree the “epidemiological conditions” is more appropriate than “environmental context.” The original sentence read: *“Our findings indicated that the sociality of white-tailed deer and various environmental contexts may lead to sustained transmission.”*

The revised sentence now reads [Lines 651-653]: “*Our findings indicated that epidemiological conditions and the sociality of white-tailed deer underpinning their proximity rates may lead to sustained transmission.*

Discussion sentence starting “We estimated sustained infections…” [Lines 630-632]– What does infection risk refer to for this conclusion. I suggest using language that matches the analysis and result that characterizes this range of infection risk

We appreciate the reviewer’s suggestion as it clarifies the messaging of the manuscript. The original sentence read: “We estimated sustained infections in wild and captive populations across a wide range of infection risks from both continuous transmission from humans and an initial transmission event.”

The revised sentence now reads [Lines 653-656]: *“We estimated sustained infections in wild and captive populations across a wide range of Force-Of-Infection rates from both continual spillover from humans and an initial spillover event.”*

Discussion sentence starting, “These results complement ongoing…” [Lines 658-661] – provide some citations for these complementary efforts

We have added requested citations to capture ongoing efforts to monitor spillover and spread of SARS-CoV-2 in white-tailed deer in North America. The original sentence read: *“These results complement ongoing, retrospective surveillance efforts across a range of captive and wild contexts by revealing the spillover risk of SARS-CoV-2 from infected humans and the risk of transmission between deer.”*

The sentence now reads [Lines 658-661]: *“These results complement ongoing, retrospective surveillance efforts across a range of captive and wild contexts by revealing the spillover risk of SARS-CoV-2 from infected humans and the risk of transmission between deer [20, 55].”*

Reviewer #2: My review is not included as an attachment:
SARS2 spillover in white-tailed deer in North America has been widespread and represents a complex challenge to our understanding of the disease ecology of the virus. The authors simulate the introduction, spread, and persistence of SARS-CoV-2 in white-tailed deer populations under four broadly generic scenarios including wild white-tailed deer and their captive conspecifics. These data are interesting and important for informing potential downstream control measures. The authors clearly explain the assumptions used in the modelling approach. The manuscript is well written and organized but is very long and should be reduced where/if possible. I only have minor comments/revisions:

Specific comments:
Lines 71-72: Authors should also include that zoonotic disease transmission is bi-directional (animal to human and human to animal).

We appreciate the reviewer’s suggestion of adding language around the bi-directional transmission of zoonotic disease. We’ve altered the first sentence of the introduction to reflect this detail. The sentence originally read: “*Many emerging infectious diseases in animal populations are transmissible to humans, representing a public health threat [1,2]*.”

The sentence now reads [Lines 71-72]: “*Many emerging infectious diseases in animal populations are transmissible between animals and humans, representing a public health threat [1,2].*”

Lines 143-145: This statement does not belong in the introduction. Please remove.

We agree that this statement is more appropriate for the discussion section. We have removed the last statement of the introduction, “*Our findings inform One Health efforts to reduce future introductions and transmission among white-tailed deer and diminish the risk of SARS-CoV-2 becoming enzootic in white-tailed deer across their North American range*.”

Lines 551-552: I think the authors meant to say “…average prevalence, probability of persistence, and incidence proportion….”

We agree that the order of these terms should match the order they are presented in the results (Figure 4). The original passage read: “*Simulated outbreaks of SARS-CoV-2 were variable across scenarios, with higher average prevalence, incidence proportions, and probability of persistence in captive scenarios relative to wild scenarios (Table 2; Fig 4). Intensive facilities had the highest average prevalence, incidence proportion, and probability of SARS-CoV-2 persistence, followed by the outdoor ranch scenario and both wild scenarios (Table 2)*.”

The revised sentence now reads [Lines 562 - 567]: “*Simulated outbreaks of SARS-CoV-2 were variable across scenarios, with higher average prevalence, probability of persistence, and incidence proportion in captive scenarios relative to wild scenarios (Table 2; Fig 4). Intensive facilities had the highest average prevalence, probability of SARS-CoV-2 persistence, and incidence proportion, followed by the outdoor ranch scenario and both wild scenarios (Table 2).*”

We have updated the order of these metrics throughout the manuscript, as there were several instances where the order was not consistent with how the results are presented.

We also updated the caption for Figure 4 to reflect this reordering, fixed an overlooked typo, and make the legend better for stand-alone interpretation. The caption originally read: “Fig 4. Box and whisker plots of average prevalence, incidence proportion, and incidence proportion values during the 120-day fall projection in each scenario of interest. Box plots depict the minimum, first quartile, median, third quartile, and maximum, with outliers depicted as single points. Error bars for persistence represent 95% confidence intervals.”

The caption now reads: “Fig 4. Distributions of average prevalence, persistence probability, and incidence proportion values during the 120-day fall projection in each scenario of interest. 1000 simulations were run for each scenario. Box and whisker plots depict the minimum, first quartile, median, third quartile, and maximum, with outliers depicted as single points. Error bars for persistence represent 95% confidence intervals.”

We also updated column order and caption titles for Table 2 and 3 to reflect this consistent ordering.

Figure 6: The way this figure is currently laid out makes the comparison across conditions hard. Would it be possible to put the conditions (e.g. wild rural, wild suburban etc) along the x-axis?

We appreciate the reviewer’s perspective with this figure. However, our goal with this plot was to illustrate the changes in outbreak metrics with continual introduction and single introduction events within each scenario. If we changed the plot as suggested, we think that this important result will be lost and will lead to readers interpreting that we are comparing across scenarios, rather than within scenarios.

The line numbers end at 602, can the authors add these in when submitting the revised version of the manuscript?

We appreciate both reviewers catching this error. We have fixed the line numbering so it runs continuously through the submission.
