# Supplementary material for: Epidemiological modeling of SARS-CoV-2 in white-tailed deer (Odocoileus virginianus) reveals conditions for introduction and widespread transmission
Source: PLoS Comput Biol. 2024 Jul 12;20(7):e1012263. doi: 10.1371/journal.pcbi.1012263 (PMC11268674; doi:10.1371/journal.pcbi.1012263)
Supplement: Attachment — Submitted filename: Epi paper response to reviewers.docx [file pcbi.1012263.s019.docx]

Dear Mr. Rosenblatt,

Thank you very much for submitting your manuscript "Epidemiological modeling of SARS-CoV-2 in white-tailed deer (Odocoileus virginianus) reveals conditions for introduction and widespread transmission" for consideration at PLOS Computational Biology. As with all papers reviewed by the journal, your manuscript was reviewed by members of the editorial board and by several independent reviewers. The reviewers appreciated the attention to an important topic. Based on the reviews, we are likely to accept this manuscript for publication, providing that you modify the manuscript according to the review recommendations.

Please prepare and submit your revised manuscript within 30 days. If you anticipate any delay, please let us know the expected resubmission date by replying to this email.

When you are ready to resubmit, please upload the following:

[1] A letter containing a detailed list of your responses to all review comments, and a description of the changes you have made in the manuscript. Please note while forming your response, if your article is accepted, you may have the opportunity to make the peer review history publicly available. The record will include editor decision letters (with reviews) and your responses to reviewer comments. If eligible, we will contact you to opt in or out

[2] Two versions of the revised manuscript: one with either highlights or tracked changes denoting where the text has been changed; the other a clean version (uploaded as the manuscript file).

Important additional instructions are given below your reviewer comments.

Thank you again for your submission to our journal. We hope that our editorial process has been constructive so far, and we welcome your feedback at any time. Please don't hesitate to contact us if you have any questions or comments.

Sincerely,

Benjamin Althouse

Academic Editor

PLOS Computational Biology

Rob De Boer

Section Editor

PLOS Computational Biology

**Response to editor**:

Dear Dr. Althouse and Dr. De Boer,

Thank you for your response to our revised submission of the manuscript, “Epidemiological modeling of SARS-CoV-2 in white-tailed deer (*Odocoileus virginianus*) reveals conditions for introduction and widespread transmission.” We have addressed the remaining, sole issue identified by one reviewer, specifically our inference of deer sociality being consequential to the sustained transmission of SARS-CoV-2. We have adjusted the passage as suggested by the reviewer, detailed below in red, italicized text after the reviewer’s comment. We also removed reference to Table S1 in the list of supporting information (Line 937). This file was removed after the last round of revisions on the manuscript.

We found the reviewers’ comments constructive, and they have helped to make the manuscript more accessible to a broader audience and the inferences clearer. We hope that you find our alterations satisfactory for final acceptance and publication.

For all authors,

Dr. Elias Rosenblatt

**Response to reviewers’ comments:**

Reviewer #1: The revisions are good and I think this manuscript is a well-done and valuable contribution with one exception remaining from the initial review:

In my view, the authors did not appropriately address this comment from the initial review: "Discussion sentence beginning “Our findings indicated…” [Line 651-653] You did not include any aspect of sociality. Some aspects were including in how you modeled transmission according to previous studies, but these were constant, as far as I could tell. Remove this conclusion because the models can’t disentangle the sociality of deer (implicit in the transmission parameterization) from any of the other rates you parameterized from literature or your expert elicitation."

This revised conclusion still implies that sociality was part of your findings. However sociality was not modeled as an explicit explanation to drive contact rates. The issue that remains is that this conclusion does not follow from your methods or results.

I have no doubts that white-tailed deer sociality contributes to transmission of SARS-CoV-2 and it would be acceptable to discuss or generate hypotheses about sociality influences transmission-relevant contact rates in white-tailed deer. Social structuring and interaction in white-tailed is a complex phenomena that has a quite a bit of accumulated literature- more on sociality than how sociality leads to contact and pathogen transmission- and one thing that is clear from this literature is that sociality is not a uniform (or low variance) phenomena in white-tailed deer.

I suggest removing the attribution of sociality as underpinning the proximity rates in this sentence. Such that is reads something like: Our findings indicated that epidemiological conditions and the proximity rates of white-tailed deer may lead to sustained transmission.

Then the authors could then chose to include a brief statement that social behaviors of white-tailed deer may (or are likely to) drive the proximity and contact rates or a longer discussion as they see fit.

*We appreciate the reviewer’s point about the limit of our inferences from this study. We accept the reviewer’s suggested rephrasing of the sentence in question.*

*The sentence originally read (L641-643): “Our findings indicated that epidemiological conditions and the sociality of white-tailed deer underpinning their proximity rates may lead to sustained transmission.”*

*The sentence now reads: “Our findings indicated that epidemiological conditions and the proximity rates of white-tailed deer may lead to sustained transmission.”*
